# Brain Photobiomodulation—Preliminary Results from Regional Cerebral Oximetry and Thermal Imaging

**DOI:** 10.3390/medicines6010011

**Published:** 2019-01-16

**Authors:** Gerhard Litscher

**Affiliations:** Research Unit for Complementary and Integrative Laser Medicine, Research Unit of Biomedical Engineering in Anesthesia and Intensive Care Medicine, and TCM Research Center Graz, Medical University of Graz, Auenbruggerplatz 39, EG19, 8036 Graz, Austria; gerhard.litscher@medunigraz.at; Tel.: +43-316-385-83907; Fax: +43-316-385-595-83907

**Keywords:** photobiomodulation, brain, LED (light emitting diode) stimulation, light therapy, wavelength, stroke, dementia, mental diseases, regional cerebral oxygen saturation, thermal imaging, LED helmet

## Abstract

A new piece of equipment for LED (light emitting diode) brain photobiomodulation is introduced. Preliminary results from regional cerebral oxygen saturation and from thermography are shown before, during and after stimulation. The procedure offers a new way to quantify the biological effects of a possible innovative therapeutic method. However further measurements are absolutely necessary.

Brain photobiomodulation (PBM) with red to near-infrared (NIR) light emitting diodes (LED) could be an innovative therapy for a variety of neurological and psychological disorders [1]. Red/NIR light can stimulate mitochondrial respiratory chain complex IV (cytochrome c oxidase) and increase ATP (adenosintriphosphate) synthesis [1,2,3]. In addition, light absorption by ion channels leads to the release of Ca2+ and to the activation of transcription factors and gene expression [1]. Brain PBM therapy could improve the metabolic capacity of neurons and is able to stimulate anti-inflammatory, anti-apoptotic and antioxidant responses as well as neurogenesis and synaptogenesis [1]. Findings suggest that PBM may enhance, for example, the frontal brain functions of older adults in a safe and cost-effective manner [4].

This article introduces a new piece of LED equipment (Figure 1) for brain photobiomodulation including preliminary results from near infrared spectroscopic measurements and thermal imaging.

The first promising basic and clinical trials concerning brain photobiomodulation have already been completed; however, there is currently still a lack of useful devices for therapeutic procedures [1,2,3,4,5,6,7,8]. Suyzeko (Shenzhen Guangyang Zhongkang Technology Limited, China) developed a prototype of such an innovative device. At the TCM (Traditional Chinese Medicine) Research Center (chairman: Gerhard Litscher) of the Medical University of Graz, the first test measurements were carried out with this construction (Figure 1). Preliminary data of this pilot measurement are presented here.

The equipment is currently based on infrared LED using a wavelength of 810 nm. This wavelength has been proven recently (2018) to be one of the best for transcranial laser/light stimulation [9]. The results were confirmed by measurements performed by our research team [5,6,7,8,10].

For the new stimulation helmet, altogether 256 LEDs with a wavelength of 810 nm were used (Figure 2). The investigations were performed with all LEDs (n = 256) in active mode (60 mW; one LED; 24 mW/cm^2^; ~ 15 W total helmet). The duration of the stimulation was 15 min. Figure 2 also shows the light transmission for a human skull (middle and right side). For further calculations for the transmission factor, see previous publications [6,7,8,9,10,11].

The measurements of the changes in regional cerebral oxygen saturation (rSO_2_) were performed using an INVOS 5100C Oximeter (Somanetics Corp., Troy, MI, USA) instrument. Near infrared spectroscopy is a noninvasive method for measuring rSO_2_ through the intact skull that has been applied successfully in basic medical research and clinical indications for many years [6]. Near-infrared light (730 and 805 nm) is emitted through the skin, and after passing different kinds of tissue (skin and bone), the returned light is detected at two distances from the light source (3 and 4 cm). Based upon this principle, the spectral absorption of blood in deeper structures (2–4 cm) can be determined and defined as the rSO_2_ [5,12]. The sensors were applied in the frontal area on the right and left sides of the brains of the healthy volunteer (see Figure 1). To minimize external light influence, the head in this area was covered with an elastic band during the recording and stimulation procedure. After a resting time of 20 minutes, the LED stimulation was switched on. The results of the three sections (before (20 min), during (15 min), and after (20 min) stimulation) are indicated in Figure 3. Note the significant increase in rSO_2_ (left and right side) during and even after transcranial LED stimulation. The changes of the temperature are shown in Figure 4.

PBM therapy was developed more than 50 years ago; however, there is still no common agreement on the parameters and protocols for its clinical application. Some research teams have recommended the use of a power density of less than 100 mW/cm^2^ and an energy density of 4 to 10 J/cm^2^ [11]. Others groups recommend as much as 50 J/cm^2^ at the tissue surface [11]. Parameters like wavelength, energy, fluence, power, irradiance, pulse mode, treatment duration, and repetition rate can be applied in a wide range. Our present preliminary results showed a clear response of cerebral rSO_2_ in relation to the LED stimulation. However, it has to be mentioned that the temperature increased significantly, and these effects have to be taken into account in further studies in detail. There is also the fact that ineffective studies in cells with high mitochondrial activity appear to be due more often to over-dosing than to under-dosing [11]. Therefore, clinical studies concerning the optimal stimulation doses are necessary.

Transcranial PBM appears promising to treat different mental diseases. Pitzschke et al. [13] also measured light propagation in different areas of Parkinson’s disease (PD)-relevant deep brain tissue during transcranial and transsphenoidal illumination (at 671 and 808 nm) of a cadaver head and modeled optical parameters of human brain tissue using Monte-Carlo simulations. This study demonstrates that it is possible to also illuminate deep brain tissues transcranially and transsphenoidally. This opens therapeutic options for sufferers of PD or other cerebral diseases necessitating light therapy [13].

There have been several investigations concerning possible adverse effects for LED PBM. For example, Moro et al. explored the effects of longer term application, up to 12 weeks, of PBM (670 nm) in normal, naïve macaque monkeys. They found no histological basis for any major biosafety concerns associated with PBM delivered by an intracranial approach [14]. Hennessy and Hamblin also pointed out the already established safety and notable lack of adverse effects of transcranial PBM [2].

The preliminary results are very promising; however, further research work is required in order to be able to use, for example, this new kind of PBM as a therapeutic method. Many investigators believe that PBM with LED and/or laser for brain disorders will become one of the most important medical applications of light therapy in the coming years and decades [3].

## Figures and Tables

**Figure 1:**
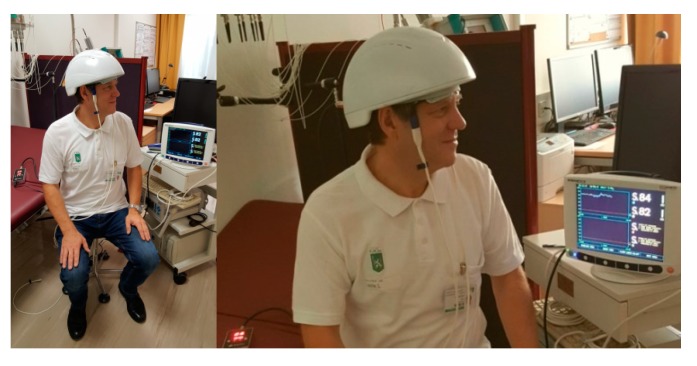
First measurement with the innovative LED (light emitting diode) photobiomodulation helmet (prototype from Suyzeko (Shenzhen Guangyang Zhongkang Technology Limited, China)) at the TCM Research Center at the Medical University of Graz, Austria, Europe performed on 25 December 2018.

**Figure 2:**
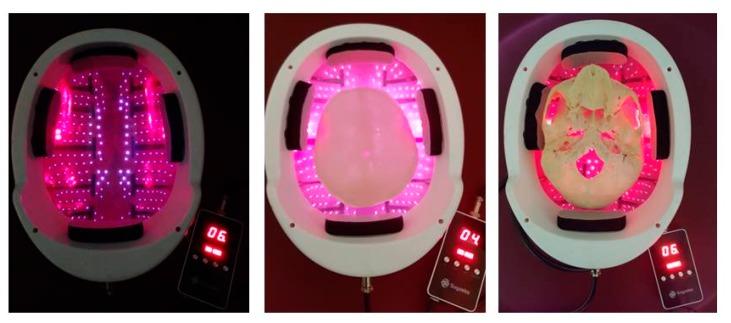
Helmet from Suyzeko (Shenzhen, China) for possible brain photobiomodulation therapy (3 January 2019).

**Figure 3:**
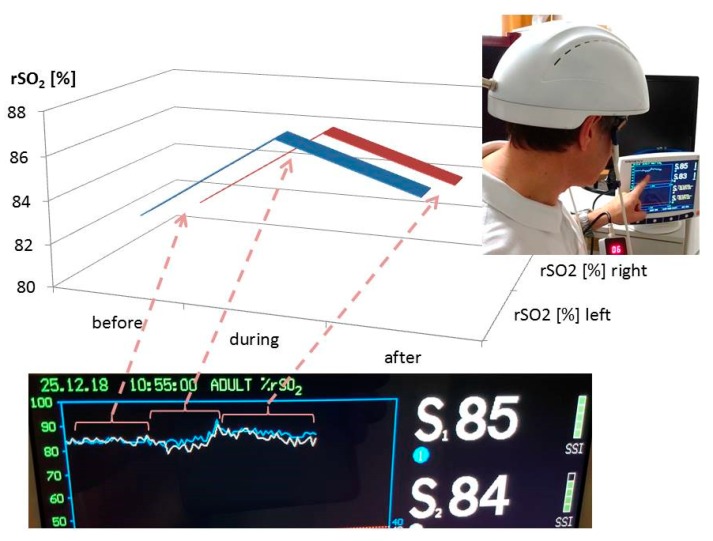
Results of the first pilot measurement with the LED stimulation helmet from Suyzeko (Shenzhen, China). Note the increase in the regional cerebral oxygen saturation during and after stimulation on the left and right side.

**Figure 4:**
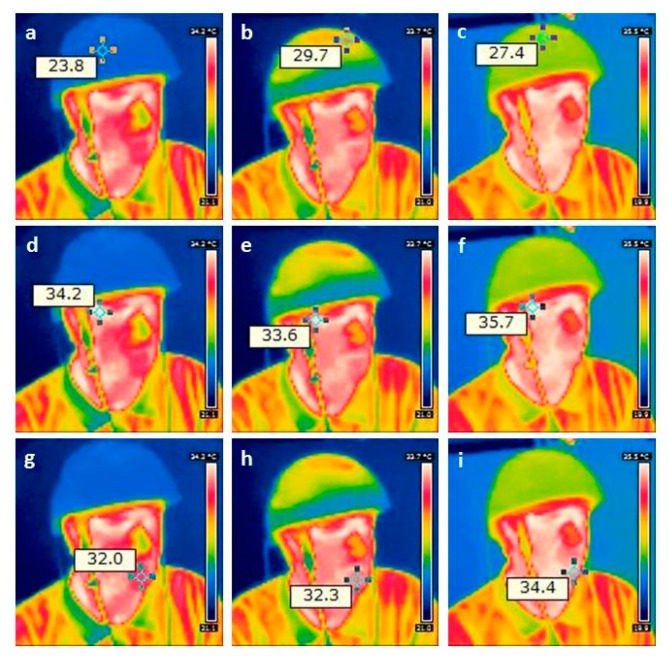
Results from thermal imaging of the first pilot measurement using the new stimulation helmet. Note the increase in temperature on the helmet (upper row; **a** before, **b** during, and **c** after stimulation) on the forehead (middle row; **d**–**f**) and on the chin (lower row; **g**–**i**).
